# Minilaparotomy a Good Option in Specific Cases: A Case Report of Bilateral Ovarian Germ Cell Tumor

**DOI:** 10.1155/2012/589568

**Published:** 2012-03-05

**Authors:** D. Bolla, N. Deseö, A. Sturm, A. Schöning, C. Leimgruber

**Affiliations:** Department of Obstetrics and Gynecology, Spitalregion Fürstenland Toggenburg, 9500 Wil, Switzerland

## Abstract

Mature cystic teratomas (MCTs) of the ovary represent 44% of ovarian neoplasmas. The surgical approach is important in young women especially for the cosmetic results. Nowadays most of the ovarian surgeries can be performed laparoscopically. An alternative between laparoscopy and laparotomy is the minilaparotomy (ML) which can be an interesting option, thanks to the small incision. We report a 39-year-old woman who was referred to our hospital with acute abdominal pain. In her past history the patient had an uncomplicated delivery. During pregnancy a 6 cm bilateral MCT was diagnosed and expectant management was followed. A left-sided ovarial torsion was postulated, and laparoscopic detorsion was performed. To avoid a rupture of the left MCT, the operation was interrupted. To remove the cyst, a ML was done two weeks later. A left-sided salpingo-oophorectomy was performed due to a large cyst including the entire ovary. On the other side, the right dermoid cyst was entirely removed. The advantage of a ML is not only shorter operating time with less learning curve compared to laparoscopy but also the possibility to extract the adnexal mass from the abdominal cavity with lower risk of rupture and in addition the possibility to preserve more ovarian tissue.

## 1. Introduction

Mature cystic teratomas (MCTs) of the ovary, also known as dermoid cysts, represent 44% of ovarian neoplasmas. They are benign tumors containing mature tissue from all of the three germ-cell layers. MCT represents up to 52% of all ovarian tumors diagnosed in women younger than 40 years. Malignant transformation is rare and occurs in 1–3% of cases [1].

The surgical approach is particularly important in young women who wish the best cosmetic results after the operation. Nowadays most surgeries for ovarian benign disease can be performed laparoscopically [2]. Another approach in special cases is the minilaparotomy (ML), which is considered by some surgeons as a minimally invasive procedure [3].

We present a case in which a woman affected from a bilateral dermoid cyst was operated with a minimal invasive procedure allowing, in this way, the preservation of their fertility.

## 2. Case Report

A 39-year-old woman was referred to our hospital with an acute abdominal pain. Five months before the time of admission, an uncomplicated spontaneous vaginal delivery occurred.

During this pregnancy, a 6 cm bilateral adnexal mass was incidentally discovered by a routine gynecological check up. The ultrasound examination and its followup described these findings as a bilateral multicystic teratoma. Because of pregnancy, an expectant management was followed.

At the time of admission, a physical and an sonographic examination was performed and a left-side ovarial torsion was suspected. A consecutive laparoscopic exploration confirmed our suspicion. A detorsion of the left adnexa was performed with a successful salvage of the ovary. The uterus was unremarkable. Both ovaries showed multiple smooth cysts of approximately 6 cm, which were identified as dermoid cysts. No ascites or tumour implants were found in the peritoneal cavity. To avoid a rupture of the left dermoid cysts, especially after an ovarial torsion, the operation was interrupted and an ML was planed.

**Figure 1:**
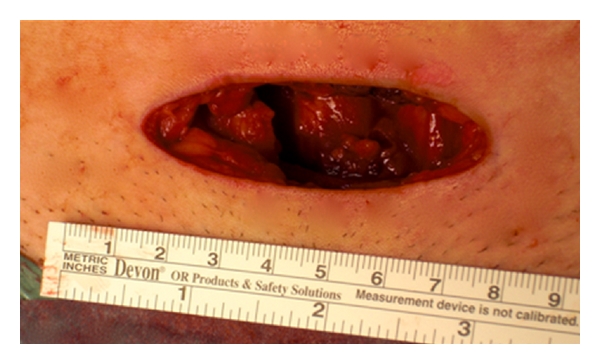
minilaparotomy (7 cm).

The ML was performed two weeks later. The suprapubic incision measuring 7 cm was close to the pubic hair line (Figure 1). The abdominal fascia was cut 2 to 3 cm above the skin incision and the peritoneum opened manually.

**Figure 2:**
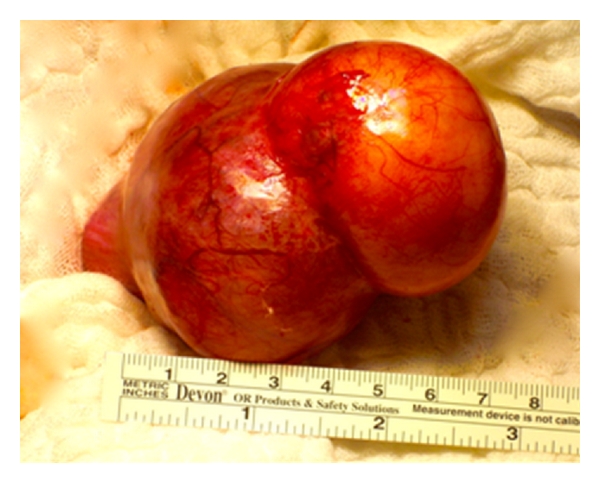
left dermoid cysts extracted from the abdominal cavity.

**Figure 3:**
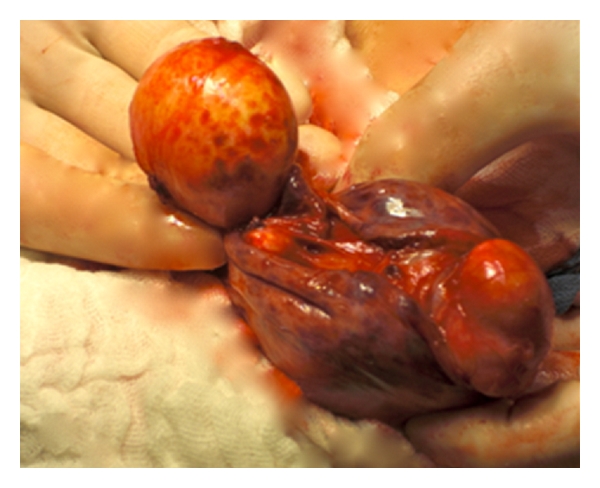
the right-sided dermoid cyst was entirely removed, allowing the preservation of the residual ovary.

**Figure 4:**
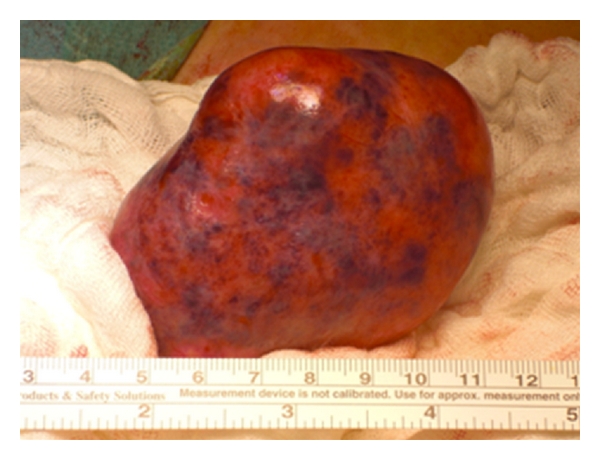
right dermoid cyst extracted from the abdominal cavity. Sterile pads were placed around the cyst to avoid, in case of a rupture, a contamination of the abdominal cavity.

The left dermoid cysts could be easily extracted from the abdominal cavity (Figure 2). A left-sided salpingo-oophorectomy was performed due to a large cyst including the entire left ovary. Fortunately the right-sided dermoid cyst was entirely removable, allowing the preservation of the residual ovary. Also the second dermoid cyst could be extracted from the abdominal cavity without complications. Sterile pads were placed around the cyst to avoid, in case of a rupture, a contamination of the abdominal cavity (Figures 3 and 4).

The patient had an uncomplicated postoperative recovery. Two month, after the operations an ultrasound was performed showing an intact ovary. After surgery no blood test for sex hormones was performed. The patient was in good general health with regular menses and without evidence of postmenopausal symptoms.

## 3. Discussion

The preservation of ovarian tissue in patients with an ovarian benign disease is considered as particularly important, especially for those in which fertility is an issue. The appropriate surgical treatment is still a matter of controversy. The laparoscopic approach to teratomas is nowadays considered as a safe and efficient procedure. In comparison to laparotomy, it does not increase complications and does not cause an increase in recovery time, hospital stay, and cost. Also the risk of adhesion formation is not as high as that in laparotomy [4]. Between laparoscopy and laparotomy another possible alternative is the minilaparotomy, which represents because of a small incision (4–9 cm) an interesting option.

If surgery is indicated for benign ovarian disease in particularly in premenopausal women, like in our case, a cystectomy or enucleation of a solid tumor from the ovary is generally considered the best alternative to preserve the ovarian tissue. If the ovary cannot be salvaged or insufficient viable tissue remains, then an oophorectomy has to be performed.

Most teratomas are cystic and composed of mature adult-type tissues. The MCT accounts for more than 95 percent of all ovarian teratomas and is almost always benign. In 10 to 15 percent of cases, dermoid cysts can be bilateral and multicystic and therefore can cause complications during surgery [5]. In approximately 1-2% of cases, a transformation into malignant tumor (usually into a squamous cell cancer) arising from one of the three germ-cell layers can occur. This happens usually up the fourth decade of life [1, 6].

During an operation a rupture of a dermoid cyst with spillage of the material into the abdominal cavity is the most common complication that can occur during both, laparoscopy (15–100%) and laparotomy (4–13%). Shock and haemorrhages are the immediate consequence of a rupture caused by chemical peritonitis (0.2%), and this induces the formation of adhesions [2]. For this reason the preoperative evaluation of the adnexal mass and the choice of operation technique are extremely important to reduce intraoperative complications and preserve ovarian tissue especially when childbearing is not completed.

This is the reason why we recommend, in cases like the presented one, a minilaparotomy, which represents an approach between laparoscopy and laparotomy. The advantage of this technique, also considered in literature as a minimal invasive procedure, is not only a shorter operating time with a less learning curve than with laparoscopy but also the possibility to remove a complex adnexal mass preserving more ovarian tissue with a reduced risk of rupture and subsequent lower risk for complications [3, 7].

In our case also a left oophorectomy was performed. In literature limited data is available about fertility following removal of one ovary. In those cases a pregnancy rate around 42 to 88 percent has been reported [8, 9]. This large difference probably is correlated to the age of the patients (younger women have more follicles per ovary) and remaining ovarian tissue after surgery.

In conclusion if a teratoma occurs, laparoscopy is considered the gold standard for its treatment. In special cases if the dermoid cyst is bilateral, multicystic, very large with a high risk of rupture and childbearing is not completed, a minilaparotomy can be a good option also because each tumor can be easily extracted from the abdominal cavity reducing complications and in this way giving the chance to preserve more ovarian tissue.
